# Effect of Vitamin D Supplementation on Cardiometabolic Outcomes in Older Australian Adults—Results from the Randomized Controlled D-Health Trial

**DOI:** 10.3390/nu18020357

**Published:** 2026-01-22

**Authors:** Briony L. Duarte Romero, Bruce K. Armstrong, Catherine Baxter, Dallas R. English, Peter R. Ebeling, Gunter Hartel, Michael G. Kimlin, Renhua Na, Donald S. A. McLeod, Hai Pham, Tanya Ross, Jolieke C. van der Pols, Alison J. Venn, Penelope M. Webb, David C. Whiteman, Rachel E. Neale, Mary Waterhouse

**Affiliations:** 1 Population Health Program, QIMR Berghofer, Brisbane 4006, Australia; 2 School of Public Health, University of Sydney, Sydney 2050, Australia; 3 Melbourne School of Population Health, University of Melbourne, Melbourne 3053, Australia; 4 Cancer Epidemiology Division, Cancer Council Victoria, Melbourne 3002, Australia; 5 Department of Medicine, School of Clinical Sciences at Monash Health, Monash University, Melbourne 3168, Australia; peter.ebeling@monash.edu; 6 Griffith Institute for Biomedicine and Glycomics, Griffith University, Gold Coast 4215, Australia; 7 School of Nursing, Faculty of Health, Queensland University of Technology, Brisbane 4059, Australia; 8 School of Biomedical Science, Faculty of Health, Queensland University of Technology, Brisbane 4059, Australia; 9 Faculty of Medicine and Medical Sciences, Bond University, Gold Coast 4226, Australia; 10 School of Public Health, The University of Queensland, Brisbane 4006, Australia; 11 Department of Endocrinology and Diabetes, Royal Brisbane and Women’s Hospital, Brisbane 4029, Australia; 12 School of Exercise and Nutrition Sciences, Faculty of Health, Queensland University of Technology, Brisbane 4059, Australia; 13 Menzies Institute for Medical Research, University of Tasmania, Hobart 7000, Australia

**Keywords:** vitamin D, diabetes, hypertension, hypercholesterolemia

## Abstract

**Background/Objectives**: Observational studies have found inverse associations between 25-hydroxyvitamin D concentration and risk of hypertension, hypercholesterolemia and type 2 diabetes (T2D). More robust evidence from large-scale randomized controlled trials, however, is limited or inconclusive. **Methods**: The D-Health Trial (N = 21,315) is a randomized, double-blind, placebo-controlled trial of supplementation with monthly doses of 60,000 international units of oral vitamin D_3_, conducted in Australians aged 60–84 years. Commencing treatment with anti-hypertensive, lipid-modifying, or anti-diabetic drugs was used as a surrogate for incident hypertension, hypercholesterolemia, and T2D, respectively. Outcomes were ascertained via linkage with the Australian Pharmaceutical Benefits Scheme database. Follow-up began 6 months after randomization; we excluded participants without linked data, and those who were prevalent cases or who died prior to start of follow-up. Flexible parametric survival models were used to estimate the effect of vitamin D supplementation on each outcome. **Results**: We included 10,964 participants (vitamin D, *n* = 5456 [49.8%]; placebo, *n* = 5508 [50.2%]) in the analysis of hypertension, 12,126 participants (vitamin D, *n* = 6038 [49.8%]; placebo, *n* = 6088 [50.2%]) in the analysis of hypercholesterolemia, and 17,846 (vitamin D, *n* = 8931 [50.0%]; placebo, *n* = 8915 [50.0%]) in the analysis of T2D. Over a median follow-up of 4.6 years, 2672 (24.4%), 2554 (21.1%), and 779 (4.4%) participants developed hypertension, hypercholesterolemia, and T2D, respectively. Vitamin D supplementation had no material effect on the incidence of any of hypertension (HR 1.00; 95% CI 0.93 to 1.08), hypercholesterolemia (HR 1.05; 95% CI 0.97 to 1.13), or T2D (HR 0.97; 95% CI 0.84 to 1.12). **Conclusions**: Monthly supplements of vitamin D did not alter the incidence of any of the three conditions in older, largely vitamin D-replete Australians.

## 1. Introduction

Hypertension, hypercholesterolemia, and type 2 diabetes (T2D) are cardiometabolic conditions that are major risk factors for cardiovascular disease and impose substantial global health and economic burdens [1,2,3,4]. Beyond its well-established role in bone metabolism and calcium homeostasis, accumulating evidence suggests that vitamin D may play an important role in cardiometabolic regulation [5]. This concept is supported by compelling biological mechanisms including effects on vascular smooth muscle function, lipid metabolism, and insulin sensitivity [6,7,8]. Vitamin D might, therefore, provide a simple and effective way to reduce the risk of these conditions in the general population.

Observational studies consistently demonstrate inverse associations between 25-hydroxyvitamin D [25(OH)D] concentration and risk of hypertension [9], dyslipidemia [10], and T2D [11]. Mendelian randomization studies provide further support for causal relationships between vitamin D status and hypertension and hypercholesterolemia, but have not supported a causal role in T2D [12,13,14]. Evidence from randomized controlled trials (RCTs) presents a conflicted picture. A meta-analysis of RCTs found no evidence that vitamin D supplementation reduces blood pressure [15]. The most comprehensive summary of results from RCTs found that vitamin D supplementation might lower triglyceride levels but it was unclear whether it had an effect on total cholesterol [16]. Similarly, results from RCTs seeking to understand the effect of vitamin D supplementation on T2D have been mixed, revealing meaningful benefits in some high-risk groups [17,18] and no effect elsewhere [19,20].

The D-Health Trial was launched to investigate the effect of supplementation with monthly doses of vitamin D on health outcomes in older Australians. The primary outcome of the trial was all-cause mortality [21]. In previous analyses of the D-Health cohort, we found that vitamin D supplementation may reduce the risk of major cardiovascular events (hazard ratio, [HR] 0.91; 95% confidence interval [CI] 0.81 to 1.01) [22], while having little effect on mortality due to cardiovascular disease (HR 0.96; 95% CI 0.72 to 1.28) [21].

In light of the currently inconsistent evidence, we aimed to determine if supplementation with monthly doses of 60,000 international units (IU) of vitamin D_3_ altered the incidence of hypertension, hypercholesterolemia, or T2D using new prescription of a relevant medication as a surrogate for diagnosis.

## 2. Materials and Methods

### 2.1. Trial Design, Participants, and Intervention

The D-Health Trial was a randomized, placebo-controlled, double-blind trial, with two parallel arms. Trial methods, including sample size justification, were published in 2016 [23], and the full trial protocol is available online (https://www.qimrb.edu.au/studies/d-health-study (accessed on 27 October 2025)). Participants were Australians aged 60 to 84 years recruited using a population register as the sampling frame. Volunteers were also recruited. To be eligible for the trial, potential participants needed to be taking no more than 500 IU/day supplemental vitamin D, and to have no self-reported history of kidney stones, hypercalcemia, hyperparathyroidism, osteomalacia, or sarcoidosis. We randomized 21,315 participants (January 2014 to May 2015) in a 1:1 ratio to monthly oral doses of either 60,000 IU of vitamin D_3_ or placebo (identical in appearance). The intervention ended 5 years after randomization, or on 1 February 2020, for participants randomized after February 2015. Randomization was stratified by age (60–64, 65–69, 70–74, ≥75 years), sex, and state of residence (New South Wales, Queensland, South Australia, Tasmania, Victoria, Western Australia) using automated computer-generated permuted block randomization. The allocation sequence was generated by an external statistician. Staff and investigators did not have access to the allocation list.

### 2.2. Baseline Characteristics

Participants completed a baseline survey that collected socio-demographic, lifestyle, and medical information. We did not measure serum 25(OH)D concentration in any participants at baseline. Instead, we predicted whether a participant’s deseasonalized baseline serum 25(OH)D concentration was ‘low’ (i.e., <50 nmol/L). The prediction model was built and internally validated using data and blood samples collected throughout the trial from a random subset of participants in the placebo group; it had an area under the receiver operating characteristic curve of 0.71 (95% CI 0.63 to 0.78) [24].

### 2.3. Outcomes

Incident hypertension, hypercholesterolemia and T2D were pre-specified tertiary outcomes of the D-Health Trial [25], with commencement of drugs used to treat these conditions used as a surrogate for diagnosis.

At baseline, participants were asked for consent to linkage of their records with those of Australia’s Pharmaceutical Benefits Scheme (PBS). The PBS dataset, which was supplied by Services Australia, records information each time a medication that qualifies for a government subsidy (including all medications relevant for this analysis) is dispensed. We used anatomic therapeutic classification (ATC) codes starting with C08, C09A, C09B, C09C, C09D, and C03A to ascertain treatment for hypertension, C10 for hypercholesterolemia, and A10 for T2D; further details are provided in Table S1.

For each outcome, we assumed that the condition was present at baseline (i.e., prevalent) if the participant had at least one prescription of a relevant medication (as defined above) dispensed within 6 months after randomization. Participants whose first prescription was more than 6 months after randomization were assumed to be newly diagnosed with the condition (i.e., incident cases).

### 2.4. Follow-Up and Eligibility

Follow-up started 6 months after randomization and ended at the earliest of: (i) first prescription of a relevant medication; (ii) date last known to be alive; (iii) 5 years and 1 month after randomization, or (iv) 29th February 2020 (for participants randomized after February 2015). Deaths were ascertained primarily via linkage to state death registries [21].

For all analyses, we excluded participants who did not give consent to linkage with the PBS dataset as their medication use was unknown. The remaining participants were eligible for inclusion if they were not classified as prevalent cases and they were alive 6 months post-randomization.

### 2.5. Monitoring Adherence and Adverse Events

Full details of adherence and adverse events in D-Health have been published [21]. Briefly, adverse events were reported via telephone or email. We also captured diagnoses of hypercalcemia, kidney stones, and hyperparathyroidism in annual surveys. We used annual surveys to ascertain the number of study capsules taken in the previous year and use of off-study supplements containing vitamin D. During the intervention period (beginning one year after recruitment started) we measured serum 25(OH)D concentrations in a random subset of participants (~800 each year).

### 2.6. Blinding

During the intervention period, all participants, investigators, study staff and analysts were blinded to group allocation. D-Health participants were informed of their study group allocation after the intervention period was complete (March 2020). Study staff, analysts, and investigators remained blinded to the allocation until analyses of the primary outcome (i.e., mortality) [21] were complete.

We wrote the code for the current study using a blinded dataset (i.e., the randomization group allocation and participant identification code had been removed, and participants had been randomly assigned to two groups of equal size). The data set with true randomization code was only provided to the analyst once all the code was verified and the statistical analysis plan (available at https://dhealth.qimrberghofer.edu.au/page/Publications/, accessed on 27 October 2025) had been saved on a read-only server.

### 2.7. Statistical Methods

Analyses were conducted in SAS version 9.4 (SAS Institute, Inc., Cary, NC, USA), R version 4.4.1 (R Foundation for Statistical Computing, Vienna, Austria), and STATA (StataCorp LLC, College Station, TX, USA) Version 18. We used a significance level of 0.05 with no adjustment for multiple testing.

We used chi-squared tests to compare baseline characteristics between people included in the main analyses and those excluded. Among participants included in the analyses, we assessed baseline balance between study groups using standardized mean differences (SMDs) [26].

Analyses of the effect of vitamin D supplementation on outcomes followed the intention-to-treat principle. Treating death as a competing risk, we used Aalen-Johansen methods to plot the cause-specific cumulative incidence of each outcome by randomization group. We used flexible parametric survival models (FPSMs) to estimate HRs (95% CIs) (Supplementary Materials—Methods). These models included randomization group, age, sex, and state of residence at randomization. To estimate overall HR, we used an FPSM that assumed proportional hazards. We allowed the HR to vary with time by fitting a second FPSM that included an interaction between randomization group and follow-up time. The significance of the interaction with time was assessed using a likelihood ratio test that compared models with and without the interaction. We also used FPSMs to estimate the difference in cause-specific standardized cumulative incidence; the cumulative incidence functions were standardized to the distribution of age, sex, and state of residence at randomization (Supplementary Materials—Methods).

We assessed associations between potential risk factors and each outcome using FPSMs that included randomization group, age at baseline, and sex. For this modelling, we assumed proportional hazards for all covariates.

### 2.8. Subgroup Analyses

We assessed whether the effect of vitamin D supplementation on each of the outcomes varied across pre-specified subgroups of: (a) age (<75; ≥75 years); (b) sex (men; women); (c) body mass index (BMI) (<25; 25 to <30; ≥30 kg/m^2^); (d) predicted baseline serum 25(OH)D concentration (<50; ≥50 nmol/L). We also assessed effect modification by prevalent hypercholesterolemia (no; yes) or prevalent hypertension (no; yes) in the hypertension and hypercholesterolemia analyses, respectively, and by both prevalent conditions in the diabetes analysis. We did not assess effect modification by prevalent diabetes due to the low number of cases. We assumed proportional hazards for all covariates in the FPSMs. The interaction between randomization group and each stratifying variable was assessed using a likelihood ratio test that compared models with and without the interaction term (Supplementary Materials—Methods).

### 2.9. Sensitivity Analysis

We conducted a sensitivity analysis using all possible classes of hypertension medications (ATC codes beginning with C02, C03, C07, C08, and C09), including those not included in the main analysis because they are also used to treat conditions other than hypertension. Since this made no meaningful difference, these results are not presented.

### 2.10. Ethics Approval and Trial Registration

The trial was approved by the QIMR Berghofer Medical Research Institute Human Research Ethics Committee and was monitored by an external Data and Safety Monitoring Board (Trial registration number Australian New Zealand Clinical Trials Registry: ACTRN12613000743763). Data linkage was approved by: ACT Health HREC, NSW Population and Health Services Research Ethics Committee, Department of Health WA HREC, and University of Tasmania HREC. Written or online informed consent was obtained from all participants.

### 2.11. Role of the Funding Source

The funding source had no role in the study design, collection, analysis, or interpretation of data, in writing the report, or in the decision to submit the manuscript for publication.

## 3. Results

**Figure 1:**
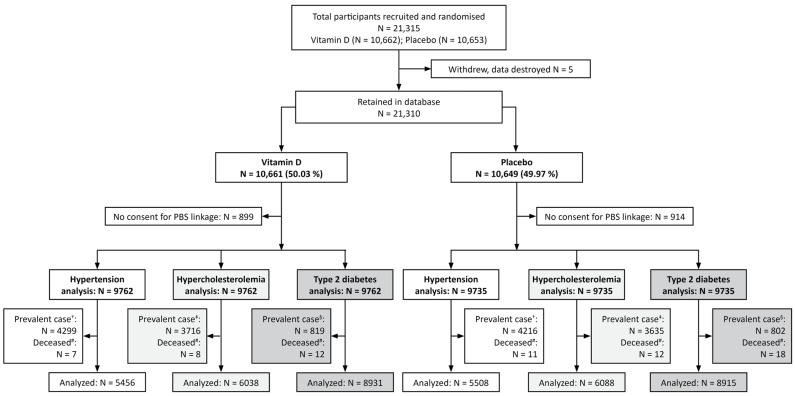
Flow of the participants included in the analyses of incident hypertension, hypercholesterolemia, and type 2 diabetes (CONSORT flow diagram). ^†^ Defined as supplied any hypertension medication within 6 months after being randomized. ^‡^ Defined as supplied any lipid-modifying agent within 6 months after being randomized. ^§^ Defined as supplied any diabetes medication within 6 months after being randomized. ^#^ Died < 6 months after randomization and not a prevalent case. Abbreviations: PBS—Pharmaceutical Benefits Scheme.

There were 19,497 (91.5%) participants with linked data. Of these, 8515 (43.7%), 7351 (37.7%), and 1621 (8.3%) participants had prevalent hypertension, prevalent hypercholesterolemia, and prevalent T2D, respectively (Figure 1). Among those who were not prevalent cases, 18 (0.2%), 20 (0.2%), and 30 (0.2%) participants were excluded from the analyses of hypertension, hypercholesterolemia, and T2D, respectively, because they died prior to the start of follow-up (Figure 1). We therefore included 10,964 participants (vitamin D, *n* = 5456 [49.8%]; placebo, *n* = 5508 [50.2%]) in the analysis of hypertension, 12,126 participants (vitamin D, *n* = 6038 [49.8%]; placebo, *n* = 6088 [50.2%]) in the analysis of hypercholesterolemia, and 17,846 (vitamin D, *n* = 8931 [50.0%]; placebo, *n* = 8915 [50.0%]) in the analysis of T2D (Figure 1). Although there were some differences in the baseline characteristics of people with and without linked data, randomization group was not associated with consent for linkage (Table S2). Similarly, randomization group was not associated with prevalent hypertension, hypercholesterolemia, or T2D (Table S2), and baseline characteristics of included participants were generally well balanced between the vitamin D and placebo groups in all analyses (Table 1).

Over a median follow-up of 4.6 years (interquartile ranges: 4.2 to 4.6 for hypertension, 4.5 to 4.6 for hypercholesterolemia, and 4.6 to 4.6 for T2D), a total of 2672 (24.4%), 2554 (21.1%), and 779 (4.4%) participants developed hypertension, hypercholesterolemia, and T2D, respectively. All three conditions were more common in people who were older, male, overweight/obese, or current or ex-smokers, and in those with predicted baseline serum 25(OH)D concentration <50 nmol/L, who did not complete school, and who had markers of poorer health (Table S3).

**Figure 2:**
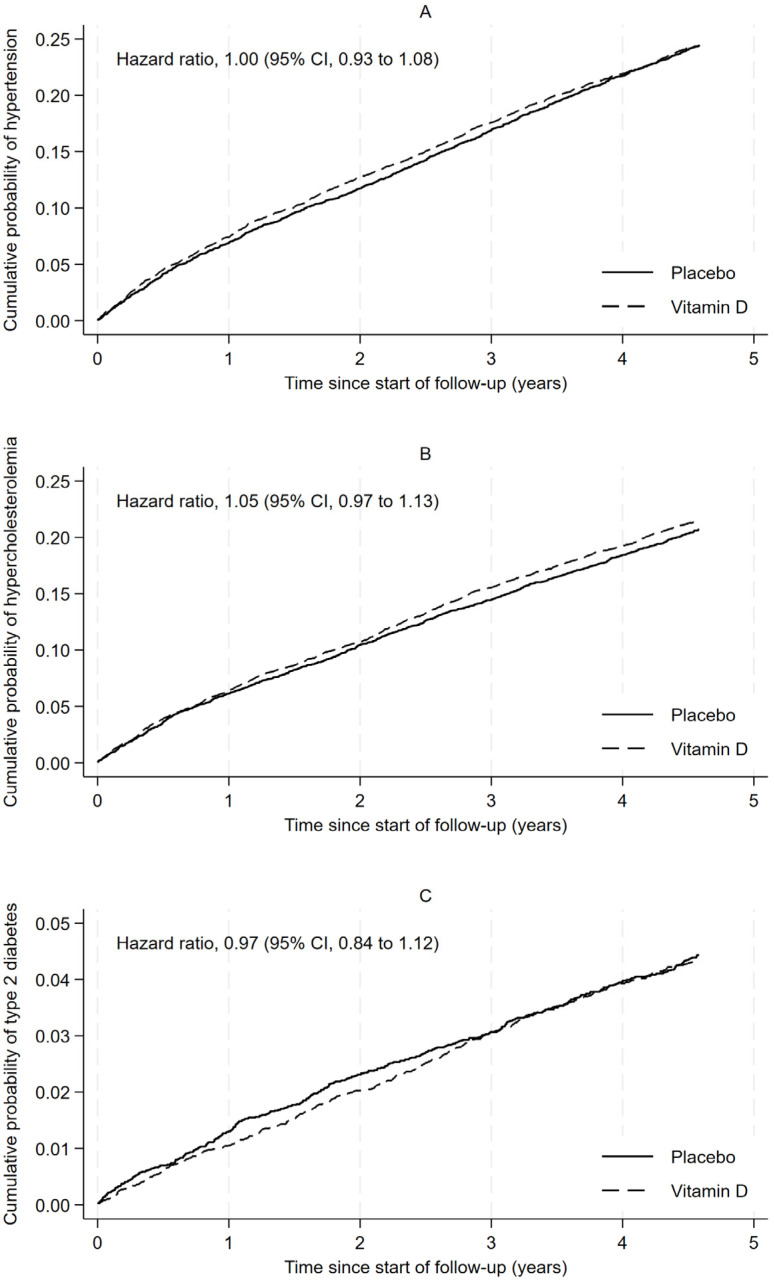
Cause-specific cumulative probability of: (**A**) incident hypertension; (**B**) incident hypercholesterolemia, and (**C**) incident diabetes according to follow-up time and randomization group. Incident hypertension, incident hypercholesterolemia and incident type 2 diabetes were defined as commencing drugs used to treat these conditions. Curves estimated using Aalen–Johansen methods, treating death without developing the condition as a competing risk. Hazard ratios (vitamin D versus placebo) were estimated using a flexible parametric survival model that included randomization group, age, sex, and state of residence at baseline. Time 0 is at 6 months after randomization, when follow-up began. People supplied with the medications within 6 months of randomization were excluded, as were participants whose last known date alive was within 6 months of randomization. Abbreviation: CI—confidence interval.

Vitamin D supplementation had a negligible effect on the overall incidence of hypertension (vitamin D, n/N = 1329/5456 [24.4%]; placebo, n/N = 1343/5508 [24.4%]; overall HR 1.00; 95% CI 0.93 to 1.08, Figure 2A), hypercholesterolemia (vitamin D, n/N = 1296/6038 [21.5%]; placebo, n/N = 1258/6088 [20.7%]; overall HR 1.05; 95% CI 0.97 to 1.13, Figure 2B), or T2D (vitamin D, n/N = 385/8931 [4.3%]; placebo, n/N = 394/8915 [4.4%]; overall HR 0.97; 95% CI 0.84 to 1.12, Figure 2C). There was little to suggest that the effect changed over time (*p*-values for the interaction between randomization group and time were 0.21, 0.92, and 0.31 for hypertension, hypercholesterolemia, and T2D, respectively; Figures S1–S3, Table S4). Similarly, there was little evidence that the effect was modified by age, sex, BMI, predicted serum 25(OH)D concentration, or prevalent disease (Figures S4–S6).

## 4. Discussion

Monthly doses of vitamin D supplementation did not reduce the incidence of hypertension, hypercholesterolemia or T2D. We also found no effect of vitamin D supplementation on these outcomes in subgroups of participants.

The incidence of hypertension, hypercholesterolemia, and T2D was 24%, 21%, and 4%, respectively, over a median 4.6 years. As expected, we saw strong associations between each condition and known risk factors such as older age and overweight/obesity [27,28,29]. Baseline prevalence of hypertension and diabetes were consistent with national estimates using self-reported survey data [30], and other similar studies which have estimated prevalence based on linked medication data [31,32]. The baseline prevalence of hyperlipidemia was markedly higher than self-reported prevalence in the general population [30]. However, similar inconsistencies have been observed elsewhere when comparing estimates based on self-reported and linked medication data [31,33]. No suitable comparison was identified to compare the incidence rate of hyperlipidemia. Incidence of hypertension and diabetes were consistent with other estimates in two similar studies [32,34].

Our finding that vitamin D supplementation did not lower the risk of hypertension is consistent with other interventional studies. In a 2020 meta-analysis of RCTs [15], vitamin D supplementation did not lower blood pressure, and two additional RCTs found no statistically significant effect on medically diagnosed hypertension [35,36]. In contrast, a Mendelian randomization study found an association between higher genetically predicted 25(OH)D concentration and lower risk of hypertension [12]. The apparent discordance between RCTs and Mendelian randomization studies might be due, in part, to differences in the type and duration of exposure [37]. Specifically, Mendelian randomization studies consider lifelong exposure to a higher concentration of 25(OH)D, whereas trials consider exposure to vitamin D supplements at a specific point in the life course, often at older ages.

We found no effect of monthly doses of supplemental vitamin D on the incidence of hypercholesterolemia. Differences in outcome measurement and study populations make direct comparison with previous RCTs challenging. A 2023 umbrella review of meta-analyses of RCTs considered the effect of vitamin D supplementation on levels of low-density lipoprotein cholesterol, high-density lipoprotein cholesterol, total cholesterol, and triglycerides [16]. The authors found that supplementation might lower triglyceride levels, but their ability to draw conclusions was hampered, in part, by the low quality of most included RCTs [16].

Vitamin D supplementation had negligible effect on T2D incidence in this cohort of older and predominantly vitamin D replete adults. This result is consistent with recent results from a similar RCT [19] and a meta-analysis of RCTs conducted in the general population [20]. In contrast, a recent meta-analysis of RCTs in high-risk (prediabetic) populations reported a 15% reduction in new onset T2D among those assigned to vitamin D supplementation [18]. The reason for this discrepancy is unclear, but it may simply be that the substantially higher incidence of T2D in high-risk groups provides greater opportunity to observe the outcome and thus better statistical power.

Our findings, and those of other RCTs, contradict the results of previous observational studies which have shown an inverse association between vitamin D levels and cardiometabolic outcomes [9,10,11]. This could reflect residual confounding by lifestyle habits or co-morbidities in observational studies. Alternatively, it may be that the specific interventions in the RCTs had no effect in the populations in which they were studied.

This study used data from the D-Health Trial, which boasts several strengths including a large sample size, and high retention and compliance (both > 80%). Ascertaining outcomes via linkage with administrative datasets has both benefits and limitations. The major benefit is complete follow-up for participants included in the analyses. With respect to limitations, we had to exclude participants who did not consent to data linkage (8.5%). However, consent to linkage was not associated with randomization group. We also did not have clinical confirmation of either prevalent or incident cases. Instead, we used very broad ATC codes to ascertain outcomes. The medications we used may have been prescribed for treatment of an unrelated condition, and we will not have ascertained cases where a participant experienced the outcome but did not receive medical treatment. Despite these limitations, for all analyses, baseline prevalence of medication use was well balanced between the vitamin D and placebo groups, and differential misclassification of cases between the treatment groups is unlikely.

Compared with the older Australian population, D-Health participants were somewhat healthier [23] and less likely to have vitamin D deficiency. Among the subset of randomly selected placebo participants who provided a blood sample, 13% had a serum 25(OH)D concentration <50 nmol/L, compared with 16% reported for Australians aged ≥65 years in the 2011–2013 Australian Health Survey [38]. We found that vitamin D supplementation was not beneficial in participants predicted to have ‘low’ 25(OH)D concentration at baseline (<50 nmol/L). However, the model we used to predict whether a participant’s baseline 25(OH)D was <50 nmol/L had a modest positive predictive value (23%), making the lack of measured baseline 25(OH)D concentrations a limitation for these stratified analyses. We cannot rule out the possibility that supplementing people with vitamin D deficiency would be beneficial for cardiometabolic outcomes. It is also important to note that the vast majority (~93%) of D-Health participants reported having British/European ancestry. Hence, we did not have sufficient power to explore effect modification by ancestry, and our findings may not be generalizable to populations with greater ethnic diversity.

## 5. Conclusions

In conclusion, monthly doses of supplemental vitamin D did not reduce the incidence of hypertension, hypercholesterolemia, or T2D in a cohort of older, generally vitamin D replete, Australians.

## Figures and Tables

**Table 1:** Baseline characteristics of the participants included in the analyses according to randomization group.

	Incident Hypertension ^≈^			Incident Hypercholesterolemia ^◎^			Incident Type 2 Diabetes ^✻^		
	N (%)			N (%)			N (%)		
Characteristic	Placebo (N = 5508)	Vitamin D (N = 5456)	SMD	Placebo (N = 6088)	Vitamin D (N = 6038)	SMD	Placebo (N = 8915)	Vitamin D (N = 8931)	SMD
**Age (years)**			0.03			0.01			0.01
60–64	1659 (30.1)	1600 (29.3)		1796 (29.5)	1771 (29.3)		2237 (25.1)	2215 (24.8)	
65–69	1596 (29.0)	1598 (29.3)		1740 (28.6)	1718 (28.5)		2475 (27.8)	2486 (27.8)	
70–74	1373 (24.9)	1344 (24.6)		1502 (24.7)	1495 (24.8)		2399 (26.9)	2416 (27.1)	
≥75	880 (16.0)	914 (16.8)		1050 (17.2)	1054 (17.5)		1804 (20.2)	1814 (20.3)	
**Sex**			<0.01			<0.01			0.01
Men	2844 (51.6)	2824 (51.8)		3110 (51.1)	3080 (51.0)		4746 (53.2)	4792 (53.7)	
Women	2664 (48.4)	2632 (48.2)		2978 (48.9)	2958 (49.0)		4169 (46.8)	4139 (46.3)	
**Body mass index (kg/m^2^)**			0.03			0.03			0.03
<25	1998 (36.3)	2051 (37.6)		2063 (33.9)	2105 (34.9)		2756 (30.9)	2877 (32.2)	
25 to <30	2362 (42.9)	2295 (42.1)		2613 (42.9)	2490 (41.2)		3897 (43.7)	3795 (42.5)	
≥30	1118 (20.3)	1089 (20.0)		1384 (22.7)	1418 (23.5)		2218 (24.9)	2225 (24.9)	
*Missing*	*30 (0.5)*	*21 (0.4)*		*28 (0.5)*	*25 (0.4)*		*44 (0.5)*	*34 (0.4)*	
**Predicted 25(OH)D concentration (nmol/L)**			0.03			0.02			0.02
<50	1291 (23.4)	1215 (22.3)		1446 (23.8)	1394 (23.1)		2122 (23.8)	2039 (22.8)	
≥50	4217 (76.6)	4241 (77.7)		4642 (76.2)	4644 (76.9)		6793 (76.2)	6892 (77.2)	
**Highest qualification obtained**			0.05			0.04			0.05
None	438 (8.0)	479 (8.8)		506 (8.3)	520 (8.6)		826 (9.3)	855 (9.6)	
School or intermediate certificate	857 (15.6)	868 (15.9)		973 (16.0)	985 (16.3)		1445 (16.2)	1492 (16.7)	
Higher school or leaving certificate	802 (14.6)	716 (13.1)		862 (14.2)	804 (13.3)		1279 (14.3)	1184 (13.3)	
Apprenticeship or certificate	1825 (33.1)	1759 (32.2)		2029 (33.3)	1935 (32.0)		2985 (33.5)	2896 (32.4)	
University degree or higher	1537 (27.9)	1575 (28.9)		1662 (27.3)	1736 (28.8)		2282 (25.6)	2416 (27.1)	
*Missing*	*49 (0.9)*	*59 (1.1)*		*56 (0.9)*	*58 (1.0)*		*98 (1.1)*	*88 (1.0)*	
**Self-rated overall health**			0.03			0.02			0.01
Excellent or very good	3440 (62.5)	3420 (62.7)		3686 (60.5)	3674 (60.8)		5084 (57.0)	5108 (57.2)	
Good	1642 (29.8)	1647 (30.2)		1936 (31.8)	1866 (30.9)		3054 (34.3)	3045 (34.1)	
Fair or poor	343 (6.2)	300 (5.5)		382 (6.3)	401 (6.6)		649 (7.3)	631 (7.1)	
*Missing*	*83 (1.5)*	*89 (1.6)*		*84 (1.4)*	*97 (1.6)*		*128 (1.4)*	*147 (1.6)*	
**Self-rated quality of life**			0.02			0.02			0.01
Excellent or very good	3898 (70.8)	3832 (70.2)		4220 (69.3)	4151 (68.7)		6074 (68.1)	6027 (67.5)	
Good	1241 (22.5)	1265 (23.2)		1455 (23.9)	1485 (24.6)		2217 (24.9)	2262 (25.3)	
Fair or poor	257 (4.7)	245 (4.5)		292 (4.8)	278 (4.6)		443 (5.0)	453 (5.1)	
*Missing*	*112 (2.0)*	*114 (2.1)*		*121 (2.0)*	*124 (2.1)*		*181 (2.0)*	*189 (2.1)*	
**Prevalent hypertension ^†^**			<0.01			0.03			0.02
No	5508 (100)	5456 (100)		4267 (70.1)	4155 (68.8)		5336 (59.9)	5266 (59.0)	
Yes	0 (0)	0 (0)		1821 (29.9)	1883 (31.2)		3579 (40.1)	3665 (41.0)	
**Prevalent hypercholesterolemia ^‡^**			0.03			<0.01			0.02
No	4267 (77.5)	4155 (76.2)		6088 (100)	6038 (100)		5925 (66.5)	5857 (65.6)	
Yes	1241 (22.5)	1301 (23.8)		0 (0)	0 (0)		2990 (33.5)	3074 (34.4)	
**Prevalent diabetes ^§^**			0.02			0.02			<0.01
No	5336 (96.9)	5266 (96.5)		5925 (97.3)	5857 (97.0)		8915 (100)	8931 (100)	
Yes	172 (3.1)	190 (3.5)		163 (2.7)	181 (3.0)		0 (0)	0 (0)	
**Smoking history**			0.04			0.04			0.03
Never	3106 (56.4)	3092 (56.7)		3433 (56.4)	3431 (56.8)		4915 (55.1)	4918 (55.1)	
Ex-smoker	2092 (38.0)	2109 (38.7)		2324 (38.2)	2337 (38.7)		3538 (39.7)	3610 (40.4)	
Current	266 (4.8)	217 (4.0)		280 (4.6)	231 (3.8)		388 (4.4)	344 (3.9)	
*Missing*	*44 (0.8)*	*38 (0.7)*		*51 (0.8)*	*39 (0.6)*		*74 (0.8)*	*59 (0.7)*	
**Alcohol consumption (drinks/week)**			0.03			0.03			0.03
<1	1250 (22.7)	1226 (22.5)		1379 (22.7)	1390 (23.0)		2020 (22.7)	1993 (22.3)	
1 to 7	2471 (44.9)	2468 (45.2)		2709 (44.5)	2669 (44.2)		3887 (43.6)	3839 (43.0)	
>7 to 14	964 (17.5)	1002 (18.4)		1066 (17.5)	1109 (18.4)		1579 (17.7)	1677 (18.8)	
>14	615 (11.2)	574 (10.5)		703 (11.5)	658 (10.9)		1106 (12.4)	1108 (12.4)	
*Missing*	*208 (3.8)*	*186 (3.4)*		*231 (3.8)*	*212 (3.5)*		*323 (3.6)*	*314 (3.5)*	

^≈^ Participants who had given consent for PBS linkage were known to be alive 6 months after randomization, and who had not had any hypertension medication prescribed within 6 months of being randomized are included in the final analysis. ^◎^ Participants who had given consent for PBS linkage were known to be alive 6 months after randomization, and who had not had any lipid-modifying agents prescribed within 6 months of being randomized are included in the final analysis. ^✻^ Participants who had given consent for PBS linkage were known to be alive 6 months after randomization, and who had not had any diabetes medication prescribed within 6 months of being randomized are included in the final analysis. ^†^ Defined as supplied hypertension medication within 6 months after being randomized. ^‡^ Defined as supplied lipid-modifying agent within 6 months after being randomized. ^§^ Defined as supplied diabetes medication within 6 months after being randomized. Abbreviations: PBS—Pharmaceutical Benefits Scheme; SMD—Standardized mean difference.

## Data Availability

The data are not available as open access because participants did not consent to their data being made available in this way.

## Supplementary Materials

The following supporting information can be downloaded at: https://www.mdpi.com/article/10.3390/nu18020357/s1, Supplementary Methods; Table S1: Anatomical Therapeutic Chemical codes for hypertension medication, lipid-modifying agents and diabetes medications [39]; Table S2: Baseline characteristics of participants included versus excluded from the final analyses; Table S3: Associations between selected baseline characteristics and incident hypertension, hypercholesterolemia and type 2 diabetes; Table S4: Effect of vitamin D supplementation on incident hypertension, hypercholesterolemia, and type 2 diabetes. Predicted difference in cause-specific standardized cumulative incidence and time-varying hazard ratio at 2 and 4 years of follow-up, and predicted overall hazard ratio; Figure S1: Effect of vitamin D supplementation on incident hypertension; Figure S2: Effect of vitamin D supplementation on incident hypercholesterolemia, Figure S3: Effect of vitamin D supplementation on incident type 2 diabetes, Figure S4: Effect of vitamin D supplementation on incident hypertension overall and within participant subgroups, Figure S5: Effect of vitamin D supplementation on incident hypercholesterolemia overall and within participant subgroups, Figure S6: Effect of vitamin D supplementation on incident type 2 diabetes overall and within participant subgroups.

## Abbreviations

The following abbreviations are used in this manuscript:

25(OH)D	25-hydroxyvitamin D
ATC	anatomic therapeutic classification
BMI	body mass index
FPSM	flexible parametric survival model
HR	Hazard ratio
IU	International unit
PBS	Pharmaceutical Benefits Scheme
RCT	randomized controlled trial
T2D	type 2 diabetes
