# Supplementary material for: Effect of Vitamin D Supplementation on Cardiometabolic Outcomes in Older Australian Adults—Results from the Randomized Controlled D-Health Trial
Source: Nutrients. 2026 Jan 22;18(2):357. doi: 10.3390/nu18020357 (PMC12845487; doi:10.3390/nu18020357)
Supplement: Supplementary file 1 [file nutrients-18-00357-s001.zip › nutrients-3979878-supplementary.pdf]

SUPPLEMENTAL MATERIALS

Effect of vitamin D supplementation on cardiometabolic outcomes in older Australian adults – results from the randomized controlled D-Health Trial

Briony L. Duarte Romero [1], Bruce K. Armstrong [2], Catherine Baxter [1], Dallas R. English [3,4], Peter R. Ebeling [5], Gunter Hartel [1,6,7], Michael G. Kimlin [8,9], Renhua Na [1,10], Donald S. A. McLeod [1,11], Hai Pham [1,10], Tanya Ross [1,10], Jolieke C. van der Pols [12], Alison J. Venn [13], Penelope M. Webb [1,10], David C. Whiteman [1,10], Rachel E. Neale [1,10,*,†], Mary Waterhouse [1,†]

1 Population Health Program, QIMR Berghofer, Brisbane 4006, Australia
2 School of Public Health, University of Sydney, Sydney 2050, Australia
3 Melbourne School of Population Health, University of Melbourne, Melbourne 3053, Australia
4 Cancer Epidemiology Division, Cancer Council Victoria, Melbourne3002, Australia
5 Department of Medicine, School of Clinical Sciences at Monash Health, Monash University, Melbourne 3168, Australia
6 Griffith Institute for Biomedicine and Glycomics, Griffith University, Queensland 4215, Australia
7 School of Nursing, Faculty of Health, Queensland University of Technology, Brisbane 4059, Australia
8 School of Biomedical Science, Faculty of Health, Queensland University of Technology, Brisbane 4059, Australia
9 Faculty of Medicine and Medical Sciences, Bond University, Gold Coast 4226, Australia
10 School of Public Health, The University of Queensland, Brisbane 4006, Australia
11 Department of Endocrinology and Diabetes, Royal Brisbane and Women's Hospital, Brisbane 4029, Australia
12 School of Exercise and Nutrition Sciences, Faculty of Health, Queensland University of Technology, Brisbane 4059, Australia
13 Menzies Institute for Medical Research, University of Tasmania, Hobart 7000, Australia
* Correspondence: Rachel.Neale@qimrb.edu.au; Tel.: +61-7-3845-3598
† These authors contributed equally to this work.

Table of Contents

Supplementary Methods – flexible parametric survival models 3

Table S1. Anatomical Therapeutic Chemical codes for hypertension medication, lipid-modifying agents and diabetes medications 4

Table S2. Baseline characteristics of participants included versus excluded from the final analyses 7

Table S3. Associations between selected baseline characteristics and incident hypertension, hypercholesterolemia and type 2 diabetes* 10

Table S4. Effect of vitamin D supplementation on incident hypertension, hypercholesterolemia, and type 2 diabetes. Predicted difference in cause-specific standardized cumulative incidence and time-varying hazard ratio at 2 and 4 years of follow-up, and predicted overall hazard ratio* 12

Figure S1. Effect of vitamin D supplementation on incident hypertension 13

Figure S2. Effect of vitamin D supplementation on incident hypercholesterolemia. 14

Figure S3. Effect of vitamin D supplementation on incident type 2 diabetes. 15

Figure S4. Effect of vitamin D supplementation on incident hypertension overall and within participant subgroups 16

Figure S5. Effect of vitamin D supplementation on incident hypercholesterolemia overall and within participant subgroups 17

Figure S6. Effect of vitamin D supplementation on incident type 2 diabetes overall and within participant subgroups 18

Supplementary Methods – flexible parametric survival models

We modelled the baseline log cumulative hazard function using a restricted cubic spline with two internal knots (placed at the 33rd and the 67th percentiles of the uncensored log time to first prescription of a relevant medication). When the model included an interaction between randomization group and follow-up time, it was fitted as a restricted cubic spline with one internal knot, placed at the median of uncensored log time to first prescription.

We used the model that included the interaction with time to produce a plot of the estimated hazard ratio and 95% confidence interval as a function of time since randomization.

We used flexible parametric survival models to predict the difference in cause-specific standardized cumulative incidence, treating death without prior outcome (i.e., hypertension, hypercholesterolemia, or type 2 diabetes) as a competing risk. For this analysis we used the user-written standsurv command in Stata with the competing risks models option.

Table S1. Anatomical Therapeutic Chemical codes for hypertension medication, lipid-modifying agents and diabetes medications

**Anatomical Therapeutic Chemical code[†]**
**A ALIMENTARY TRACT AND METABOLISM**
**A10 DRUGS USED IN DIABETES**
**A10A INSULINS AND ANALOGUES**
A10AB - Insulins and analogues for injection, fast-acting
A10AC - Insulins and analogues for injection, intermediate-acting
A10AD - Insulins and analogues for injection, intermediate- or long-acting combined with fast-acting
A10AE - Insulins and analogues for injection, long-acting
A10AF - Insulins and analogues for inhalation[‡]
**A10B BLOOD GLUCOSE LOWERING DRUGS, EXCL. INSULINS**
A10BA – Biguanides
A10BB - Sulfonylureas
A10BC - Sulfonamides (heterocyclic) [‡]
A10BD - Combinations of oral blood glucose lowering drugs
A10BF - Alpha glucosidase inhibitors
A10BG - Thiazolidinediones
A10BH - Dipeptidyl peptidase 4 (DPP-4) inhibitors
A10BJ - Glucagon-like peptide-1 (GLP-1) analogues
A10BK - Sodium-glucose co-transporter 2 (SGLT2) inhibitors
A10BX - Other blood glucose lowering drugs, excl. insulins
**A10X OTHER DRUGS USED IN DIABETES[‡]**
**C CARDIOVASCULAR SYSTEM**
**C02 ANTIHYPERTENSIVES2[¥]**
**CO2A ANTIADRENERGIC AGENTS, CENTRALLY ACTING**
C02AA- Rauwolfia alkaloids[‡]
C02AB- Methyldopa
C02AC- Imidazoline receptor agonists
**C02B ANTIADRENERGIC AGENTS, GANGLION-BLOCKING[‡]**
**C02C ANTIADRENERGIC AGENTS, PERIPHERALLY ACTING**
C02CA- Alpha-adrenoreceptor antagonists
C02CC- Guanidine derivatives[‡]
**C02D ARTERIOLAR SMOOTH MUSCLE, AGENTS ACTING ON**
C02DA - Thiazide derivatives[‡]
C02DB - Hydrazinophthalazine derivatives
C02DC - Pyrimidine derivatives
C02DD - Nitroferricyanide derivatives[‡]
C02DG - Guanidine derivatives[‡]
**C02K OTHER ANTIHYPERTENSIVES**
C02KA - Alkaloids, excl. rauwolfia[‡]
C02KB - Tyrosine hydroxylase inhibitors[‡]
C02KC - MAO inhibitors[‡]
C02KD - Serotonin antagonists[‡]
C02KN - Other antihypertensives[‡]
C02KX - Antihypertensives for pulmonary arterial hypertension
**C02L ANTIHYPERTENSIVES AND DIURETICS IN COMBINATION[‡]**
**C02N COMBINATIONS OF ANTIHYPERTENSIVES IN ATC GR. C02[‡]**
**C03 DIURETICS[¥]**

C03A LOW-CEILING DIURETICS, THIAZIDES

- C03AA - Thiazides, plain
- C03AB - Thiazides and potassium in combination[‡]
- C03AH - Thiazides, combinations with psycholeptics and/or analgesics[‡]
- C03AX - Thiazides, combinations with other drugs[‡]

C03B LOW-CEILING DIURETICS, EXCL. THIAZIDES

- C03BA - Sulfonamides, plain
- C03BB - Sulfonamides and potassium in combination[‡]
- C03BC - Mercurial diuretics[‡]
- C03BD - Xanthine derivatives[‡]
- C03BK - Sulfonamides, combinations with other drugs[‡]
- C03BX - Other low-ceiling diuretics[‡]

C03C HIGH-CEILING DIURETICS

- C03CA - Sulfonamides, plain
- C03CB - Sulfonamides and potassium in combination[‡]
- C03CC - Aryloxyacetic acid derivatives
- C03CD - Pyrazolone derivatives[‡]
- C03CX - Other high-ceiling diuretics[‡]

C03D ALDOSTERONE ANTAGONISTS AND OTHER POTASSIUM-SPARING AGENTS

- C03DA - Aldosterone antagonists
- C03DB - Other potassium-sparing agents[‡]

C03E DIURETICS AND POTASSIUM-SPARING AGENTS IN COMBINATION

- C03EA - Low-ceiling diuretics and potassium-sparing agents
- C03EB - High-ceiling diuretics and potassium-sparing agents[‡]

C03X OTHER DIURETICS

- C03XA - Vasopressin antagonists

C07 BETA BLOCKING AGENTS[¥]

C07A BETA BLOCKING AGENTS

- C07AA - Beta blocking agents, non-selective
- C07AB - Beta blocking agents, selective
- C07AG - Alpha and beta blocking agents

C07B BETA BLOCKING AGENTS AND THIAZIDES[‡]

C07C BETA BLOCKING AGENTS AND OTHER DIURETICS[‡]

C07D BETA BLOCKING AGENTS, THIAZIDES AND OTHER DIURETICS[‡]

C07E BETA BLOCKING AGENTS AND VASODILATORS[‡]

C07F BETA BLOCKING AGENTS, OTHER COMBINATIONS[‡]

C08 CALCIUM CHANNEL BLOCKERS

C08C SELECTIVE CALCIUM CHANNEL BLOCKERS WITH MAINLY VASCULAR EFFECTS

- C08CA - Dihydropyridine derivatives
- C08CX - Other selective calcium channel blockers with mainly vascular effects[‡]

C08D SELECTIVE CALCIUM CHANNEL BLOCKERS WITH DIRECT CARDIAC EFFECTS

- C08DA - Phenylalkylamine derivatives
- C08DB - Benzothiazepine derivatives

C08E NON-SELECTIVE CALCIUM CHANNEL BLOCKERS

- C08EA - Phenylalkylamine derivatives[‡]
- C08EX - Other non-selective calcium channel blockers

C08G CALCIUM CHANNEL BLOCKERS AND DIURETICS[‡]

C09 AGENTS ACTING ON THE RENIN-ANGIOTENSIN SYSTEM

C09A ACE INHIBITORS, PLAIN

- C09AA - ACE inhibitors, plain

C09B ACE INHIBITORS, COMBINATIONS

		C09BA - ACE inhibitors and diuretics
		C09BB - ACE inhibitors and calcium channel blockers
		C09BX - ACE inhibitors, other combinations‡
	**C09C ANGIOTENSIN II RECEPTOR BLOCKERS (ARBs), PLAIN**	
		C09CA - Angiotensin II receptor blockers (ARBs), plain
	**C09D ANGIOTENSIN II RECEPTOR BLOCKERS (ARBs), COMBINATIONS**	
		C09DA - Angiotensin II receptor blockers (ARBs) and diuretics
		C09DB - Angiotensin II receptor blockers (ARBs) and calcium channel blockers
		C09DX - Angiotensin II receptor blockers (ARBs), other combinations
	**C09X OTHER AGENTS ACTING ON THE RENIN-ANGIOTENSIN SYSTEM¥‡**	
**C10 LIPID-MODIFYING AGENTS**		
	**C10A LIPID MODIFYING AGENTS, PLAIN**	
		C10AA - HMG CoA reductase inhibitors
		C10AB - Fibrates
		C10AC - Bile acid sequestrants
		C10AD - Nicotinic acid and derivatives‡
		C10AX - Other lipid modifying agents
	**C10B LIPID MODIFYING AGENTS, COMBINATIONS**	
		C10BA - Combinations of various lipid modifying agents
		C10BX - Lipid modifying agents in combination with other drugs

† Source: https://atcddd.fhi.no/atc_ddd_index/ [1]

‡ These medications did not appear during the follow-up period.

¥ Used in the sensitivity analyses only (data not shown).

Table S2. Baseline characteristics of participants included versus excluded from the final analyses

	Consent to PBS linkage			Incident hypertension[≈]			Incident hypercholesterolemia[©]			Incident type 2 diabetes[✳]		
**Characteristic**	**Yes N (%)**	**No N (%)**	**P-value[¥]**	**Included N (%)**	**Excluded N (%)**	**P-value[¥]**	**Included N (%)**	**Excluded N (%)**	**P-value[¥]**	**Included N (%)**	**Excluded N (%)**	**P-value[‡]**
**Randomization group**												
Placebo	9735 (49.9)	914 (50.4)	0.69	5508 (50.2)	4227 (49.5)	0.33	6088 (50.2)	3647 (49.5)	0.32	8915 (50.0)	820 (49.7)	0.82
Vitamin D	9762 (50.1)	899 (49.6)		5456 (49.8)	4306 (50.5)		6038 (49.8)	3724 (50.5)		8931 (50.0)	831 (50.3)	
**Age (years)**												
60-64	4786 (24.5)	466 (25.7)	0.22	3259 (29.7)	1527 (17.9)	<0.01	3567 (29.4)	1219 (16.5)	<0.01	4452 (24.9)	334 (20.2)	<0.01
65-69	5367 (27.5)	467 (25.8)		3194 (29.1)	2173 (25.5)		3458 (28.5)	1909 (25.9)		4961 (27.8)	406 (24.6)	
70-74	5314 (27.3)	482 (26.6)		2717 (24.8)	2597 (30.4)		2997 (24.7)	2317 (31.4)		4815 (27.0)	499 (30.2)	
≥ 75	4030 (20.7)	398 (22.0)		1794 (16.4)	2236 (26.2)		2104 (17.4)	1926 (26.1)		3618 (20.3)	412 (25.0)	
**Sex**												
Men	10663 (54.7)	867 (47.8)	<0.01	5668 (51.7)	4995 (58.5)	<0.01	6190 (51.0)	4473 (60.7)	<0.01	9538 (53.4)	1125 (68.1)	<0.01
Women	8834 (45.3)	946 (52.2)		5296 (48.3)	3538 (41.5)		5936 (49.0)	2898 (39.3)		8308 (46.6)	526 (31.9)	
**Predicted 25(OH)D concentration (nmol/l)**												
< 50	4718 (24.2)	482 (26.6)	0.02	2506 (22.9)	2212 (25.9)	<0.01	2840 (23.4)	1878 (25.5)	<0.01	4161 (23.3)	557 (33.7)	<0.01
≥ 50	14779 (75.8)	1331 (73.4)		8458 (77.1)	6321 (74.1)		9286 (76.6)	5493 (74.5)		13685 (76.7)	1094 (66.3)	
**State of residence**												
Queensland	3893 (20.0)	313 (17.3)	0.03	2237 (20.4)	1656 (19.4)	0.02	2369 (19.5)	1524 (20.7)	<0.01	3568 (20.0)	325 (19.7)	0.60
New South Wales	3989 (20.5)	353 (19.5)		2199 (20.1)	1790 (21.0)		2414 (19.9)	1575 (21.4)		3627 (20.3)	362 (21.9)	
Victoria	3370 (17.3)	332 (18.3)		1826 (16.7)	1544 (18.1)		2095 (17.3)	1275 (17.3)		3087 (17.3)	283 (17.1)	
Tasmania	2282 (11.7)	230 (12.7)		1293 (11.8)	989 (11.6)		1497 (12.3)	785 (10.6)		2083 (11.7)	199 (12.1)	
South Australia	2855 (14.6)	266 (14.7)		1616 (14.7)	1239 (14.5)		1804 (14.9)	1051 (14.3)		2618 (14.7)	237 (14.4)	
Western Australia	3108 (15.9)	319 (17.6)		1793 (16.4)	1315 (15.4)		1947 (16.1)	1161 (15.8)		2863 (16.0)	245 (14.8)	
**Highest qualification obtained**												
None	1907 (9.9)	237 (13.4)	<0.01	917 (8.4)	990 (11.7)	<0.01	1026 (8.5)	881 (12.1)	<0.01	1681 (9.5)	226 (13.9)	<0.01
School or intermediate certificate	3230 (16.7)	325 (18.4)		1725 (15.9)	1505 (17.9)		1958 (16.3)	1272 (17.5)		2937 (16.6)	293 (18.0)	
Higher school or leaving certificate	2700 (14.0)	265 (15.0)		1518 (14.0)	1182 (14.0)		1666 (13.9)	1034 (14.2)		2463 (13.9)	237 (14.6)	
Apprenticeship or certificate	6437 (33.4)	595 (33.7)		3584 (33.0)	2853 (33.9)		3964 (33.0)	2473 (34.0)		5881 (33.3)	556 (34.2)	
University degree or higher	5010 (26.0)	341 (19.3)		3112 (28.7)	1898 (22.5)		3398 (28.3)	1612 (22.2)		4698 (26.6)	312 (19.2)	
Missing	*213*	*50*		*108*	*105*		*114*	*99*		*186*	*27*	

	Consent to PBS linkage			Incident hypertension[≈]			Incident hypercholesterolemia[©]			Incident type 2 diabetes[*]		
**Characteristic**	**Yes N (%)**	**No N (%)**	**P-value[¥]**	**Included N (%)**	**Excluded N (%)**	**P-value[¥]**	**Included N (%)**	**Excluded N (%)**	**P-value[¥]**	**Included N (%)**	**Excluded N (%)**	**P-value[‡]**
**Living alone**												
No	15501 (79.9)	1387 (77.2)	0.01	8754 (80.3)	6747 (79.5)	0.18	9632 (79.9)	5869 (80.0)	0.81	14243 (80.2)	1258 (76.7)	<0.01
Yes	3896 (20.1)	409 (22.8)		2154 (19.7)	1742 (20.5)		2429 (20.1)	1467 (20.0)		3513 (19.8)	383 (23.3)	
Missing	*100*	*17*		*56*	*44*		*65*	*35*		*90*	*10*	
**Self-rated overall health**												
Excellent or very good	10719 (55.8)	928 (52.4)	0.02	6860 (63.6)	3859 (45.9)	<0.01	7360 (61.6)	3359 (46.3)	<0.01	10192 (58.0)	527 (32.5)	<0.01
Good	6838 (35.6)	680 (38.4)		3289 (30.5)	3549 (42.2)		3802 (31.8)	3036 (41.9)		6099 (34.7)	739 (45.5)	
Fair or poor	1638 (8.5)	162 (9.2)		643 (6.0)	995 (11.8)		783 (6.6)	855 (11.8)		1280 (7.3)	358 (22.0)	
Missing	*302*	*43*		*172*	*130*		*181*	*121*		*275*	*27*	
**Self-rated quality of life**												
Excellent or very good	12903 (67.7)	1057 (60.7)	<0.01	7730 (72.0)	5173 (62.1)	<0.01	8371 (70.5)	4532 (63.0)	<0.01	12101 (69.2)	802 (50.2)	<0.01
Good	5075 (26.6)	556 (31.9)		2506 (23.3)	2569 (30.8)		2940 (24.7)	2135 (29.7)		4479 (25.6)	596 (37.3)	
Fair or poor	1095 (5.7)	129 (7.4)		502 (4.7)	593 (7.1)		570 (4.8)	525 (7.3)		896 (5.1)	199 (12.5)	
Missing	*424*	*71*		*226*	*198*		*245*	*179*		*370*	*54*	
**Smoking history**												
Never	10568 (54.6)	1024 (57.4)	0.04	6198 (57.0)	4370 (51.7)	<0.01	6864 (57.0)	3704 (50.7)	<0.01	9833 (55.5)	735 (45.2)	<0.01
Ex-smoker	7957 (41.1)	680 (38.1)		4201 (38.6)	3756 (44.4)		4661 (38.7)	3296 (45.1)		7148 (40.4)	809 (49.7)	
Current	815 (4.2)	81 (4.5)		483 (4.4)	332 (3.9)		511 (4.2)	304 (4.2)		732 (4.1)	83 (5.1)	
Missing	*157*	*28*		*82*	*75*		*90*	*67*		*133*	*24*	
**Alcohol consumption (drinks/week)**												
< 1.0	4606 (24.5)	441 (25.7)	0.03	2476 (23.4)	2130 (26.0)	<0.01	2769 (23.7)	1837 (25.9)	<0.01	4013 (23.3)	593 (38.0)	<0.01
1.0-7.0	8310 (44.3)	794 (46.3)		4939 (46.7)	3371 (41.1)		5378 (46.0)	2932 (41.4)		7726 (44.9)	584 (37.4)	
7.1-14.0	3457 (18.4)	297 (17.3)		1966 (18.6)	1491 (18.2)		2175 (18.6)	1282 (18.1)		3256 (18.9)	201 (12.9)	
> 14.0	2398 (12.8)	182 (10.6)		1189 (11.2)	1209 (14.7)		1361 (11.6)	1037 (14.6)		2214 (12.9)	184 (11.8)	
Missing	*726*	*99*		*394*	*332*		*443*	*283*		*637*	*89*	
**Prevalent hypertension[†]**												
No	..	..	..	10,964 (100.0)	18 (0.2)	<0.01	8422 (69.5)	2560 (34.7)	<0.01	10,602 (59.4)	380 (23.0)	<0.01
Yes	..	..		0 (0.0)	8515 (99.8)		3704 (30.5)	4811 (65.3)		7244 (40.6)	1271 (77.0)	
**Prevalent hypercholesterolemia[‡]**												
No	..	..	..	8422 (76.8)	3724 (43.6)	<0.01	12,126 (100.0)	20 (0.3)	<0.01	11,782 (66.0)	364 (22.0)	<0.01
Yes	..	..		2542 (23.2)	4809 (56.4)		0 (0.0)	7351 (99.7)		6064 (34.0)	1287 (78.0)	

Characteristic	Consent to PBS linkage Yes N (%)	No N (%)	P-value[¥]	Incident hypertension[≈] Included N (%)	Excluded N (%)	P-value[¥]	Incident hypercholesterolemia[©] Included N (%)	Excluded N (%)	P-value[¥]	Incident type 2 diabetes[*] Included N (%)	Excluded N (%)	P-value[‡]
**Prevalent diabetes[§]**												
No	..	..	..	10,602 (96.7)	7274 (85.2)	<0.01	11,782 (97.2)	6094 (82.7)	<0.01	17,846 (100.0)	30 (1.8)	<0.01
Yes	..	..		362 (3.3)	1259 (14.8)		344 (2.8)	1277 (17.3)		0 (0.0)	1621 (98.2)	

≈ Among participants with PBS linkage, those who had prevalent hypertension (i.e., were supplied hypertension medication within 6 months of being randomized), or who died within 6 months of randomization were ineligible for the analysis of incident hypertension

© Among participants with PBS linkage, those who had prevalent hypercholesterolemia (i.e., were supplied lipid-modifying agents within 6 months of being randomized), or who died within 6 months of randomization were ineligible for the analysis of incident hypercholesterolemia

* Among participants with PBS linkage, those who had prevalent type 2 diabetes (i.e., were supplied diabetes medication within 6 months of being randomized), or who died within 6 months of randomization were ineligible for the analysis of incident diabetes

¥ p-value from chi-squared test

† Defined as supplied any hypertension medication within 6 months after being randomized

‡ Defined as supplied any lipid-modifying agent within 6 months after being randomized

§ Defined as supplied any diabetes medication within 6 months after being randomized

Abbreviations: PBS, Pharmaceutical Benefits Scheme

Table S3. Associations between selected baseline characteristics and incident hypertension, hypercholesterolemia and type 2 diabetes*

Characteristic	Incident hypertension			Incident hypercholesterolemia			Incident type 2 diabetes		
	N/person-years	IR per 1000 person-years	HR (95% CI)	N/person-years	IR per 1000 person-years	HR (95% CI)	N/person-years	IR per 1000 person-years	HR (95% CI)
**All participants**	**2672/42,934**	**62.2**		**2554/48,371**	**52.8**		**779/78,648**	**9.9**	
**Age (years)**									
60-64	637/13,323	47.8	ref.	665/14,606	45.5	ref.	162/19,862	8.2	ref.
65-69	752/12,618	59.6	1.24 (1.12, 1.38)	771/13,744	56.1	1.23 (1.11, 1.36)	225/21,933	10.3	1.25 (1.02, 1.52)
70-74	748/10,420	71.8	1.48 (1.33, 1.65)	640/11,892	53.8	1.15 (1.03, 1.29)	225/21,175	10.6	1.23 (1.01, 1.51)
≥ 75	535/6573	81.4	1.67 (1.48, 1.87)	478/8129	58.8	1.24 (1.10, 1.39)	167/15,678	10.7	1.19 (0.96, 1.48)
**Sex**									
Women	1220/21,009	58.1	ref.	1112/24,118	46.1	ref.	266/36,998	7.2	ref.
Men	1452/21,926	66.2	1.08 (1.00, 1.17)	1442/24,253	59.5	1.27 (1.18, 1.38)	513/41,650	12.3	1.69 (1.46, 1.97)
**Body mass index (kg/m²)**									
< 25	798/16,407	48.6	ref.	734/17,055	43.0	ref.	103/25,192	4.1	ref.
25 to < 30	1187/18,142	65.4	1.33 (1.22, 1.46)	1117/20,294	55.0	1.24 (1.13, 1.36)	277/34,109	8.1	1.84 (1.47, 2.31)
≥ 30	671/8205	81.8	1.70 (1.54, 1.89)	690/10,823	63.8	1.49 (1.34, 1.65)	399/19,012	21.0	5.14 (4.14, 6.39)
**Predicted 25(OH)D concentration (nmol/l)**									
≥ 50	2048/33,225	61.6	ref.	1917/37,260	51.4	ref.	553/60,587	9.1	ref.
< 50	624/9709	64.3	1.06 (0.97, 1.16)	637/11,111	57.3	1.14 (1.05, 1.25)	226/18,061	12.5	1.44 (1.23, 1.68)
**Self-rated overall health**									
Excellent or very good	1470/27,539	53.4	ref.	1353/30,041	45.0	ref.	299/45,492	6.6	ref.
Good	933/12,462	74.9	1.38 (1.28, 1.50)	926/14,798	62.6	1.37 (1.26, 1.48)	357/26,610	13.4	1.98 (1.70, 2.31)
Fair or poor	214/2280	93.9	1.74 (1.50, 2.00)	233/2833	82.3	1.78 (1.55, 2.05)	104/5355	19.4	2.88 (2.30, 3.60)
**Self-rated quality of life**									
Excellent or very good	1769/30,706	57.6	ref.	1653/33,831	48.9	ref.	442/53,773	8.2	ref.
Good	686/9561	71.7	1.22 (1.12, 1.34)	691/11,498	60.1	1.21 (1.11, 1.32)	263/19,481	13.5	1.62 (1.39, 1.88)
Fair or poor	154/1824	84.4	1.43 (1.21, 1.68)	149/2113	70.5	1.43 (1.21, 1.69)	51/3795	13.4	1.62 (1.21, 2.17)
**Smoking history**									
Never	1414/24,598	57.5	ref.	1321/27,834	47.5	ref.	399/43,619	9.1	ref.
Ex-smoker	1127/16,147	69.8	1.19 (1.10, 1.28)	1081/18,260	59.2	1.20 (1.11, 1.31)	329/31,363	10.5	1.05 (0.91, 1.22)
Current	115/1857	61.9	1.13 (0.93, 1.37)	126/1941	64.9	1.36 (1.13, 1.64)	43/3084	13.9	1.49 (1.08, 2.04)

	Incident hypertension			Incident hypercholesterolemia			Incident type 2 diabetes		
**Characteristic**	**N/person-years**	**IR per 1000 person-years**	**HR (95% CI)**	**N/person-years**	**IR per 1000 person-years**	**HR (95% CI)**	**N/person-years**	**IR per 1000 person-years**	**HR (95% CI)**
**Highest qualification obtained**									
None	278/3416	81.4	ref.	275/3956	69.5	ref.	81/7308	11.1	ref.
School or intermediate certificate	406/6802	59.7	0.77 (0.66, 0.89)	397/7829	50.7	0.76 (0.65, 0.89)	119/12,981	9.2	0.87 (0.65, 1.15)
Higher school or leaving certificate	393/5878	66.9	0.88 (0.76, 1.03)	347/6690	51.9	0.77 (0.66, 0.91)	112/10,845	10.3	0.96 (0.72, 1.28)
Apprenticeship or certificate	904/13,971	64.7	0.83 (0.72, 0.95)	876/15,703	55.8	0.80 (0.70, 0.92)	302/25,793	11.7	1.01 (0.79, 1.29)
University degree or higher	668/12,455	53.6	0.71 (0.61, 0.81)	637/13,742	46.4	0.68 (0.59, 0.78)	157/20,905	7.5	0.66 (0.51, 0.87)

* Hazard ratios were estimated using flexible parametric survival models with adjustment for randomization group, and age and sex at baseline. Proportional hazards assumed for all covariates.

Abbreviations: CI, confidence interval; HR, hazard ratio, IR, incidence rate; ref., reference group

Table S4. Effect of vitamin D supplementation on incident hypertension, hypercholesterolemia, and type 2 diabetes. Predicted difference in cause-specific standardized cumulative incidence and time-varying hazard ratio at 2 and 4 years of follow-up, and predicted overall hazard ratio*

Years of follow-up	% Difference in Cumulative Incidence (95% CI)	Hazard Ratio (95% CI)
Incident hypertension		
2	0.66 (-0.47 to 1.79)	0.98 (0.90 to 1.06)
4	0.16 (-1.30 to 1.61)	0.93 (0.83 to 1.05)
Overall Hazard Ratio		1.00 (0.93 to 1.08)
Incident hypercholesterolemia		
2	0.58 (-0.44 to 1.59)	1.04 (0.96 to 1.13)
4	0.77 (-0.55 to 2.08)	1.03 (0.92 to 1.16)
Overall Hazard Ratio		1.05 (0.97 to 1.13)
Incident type 2 diabetes		
2	-0.19 (-0.59 to 0.20)	1.01 (0.87 to 1.17)
4	-0.15 (-0.69 to 0.39)	1.04 (0.84 to 1.30)
Overall Hazard Ratio		0.97 (0.84 to 1.12)

* Estimates (comparing vitamin D to placebo) are from flexible parametric survival models that include randomization group, age, sex, and state of residence at baseline. Time-varying estimates (i.e., estimates at 2 and 4 year of follow-up) were predicted using a model that also included an interaction between randomization group and follow-up time. Cause-specific standardized cumulative incidence was estimated treating death (without prior medication use) as a competing risk, and probabilities were standardized to the distribution of age, sex, and state of residence at baseline in the entire cohort. The difference in cumulative incidence is expressed as a percentage.

Abbreviation: CI – confidence interval

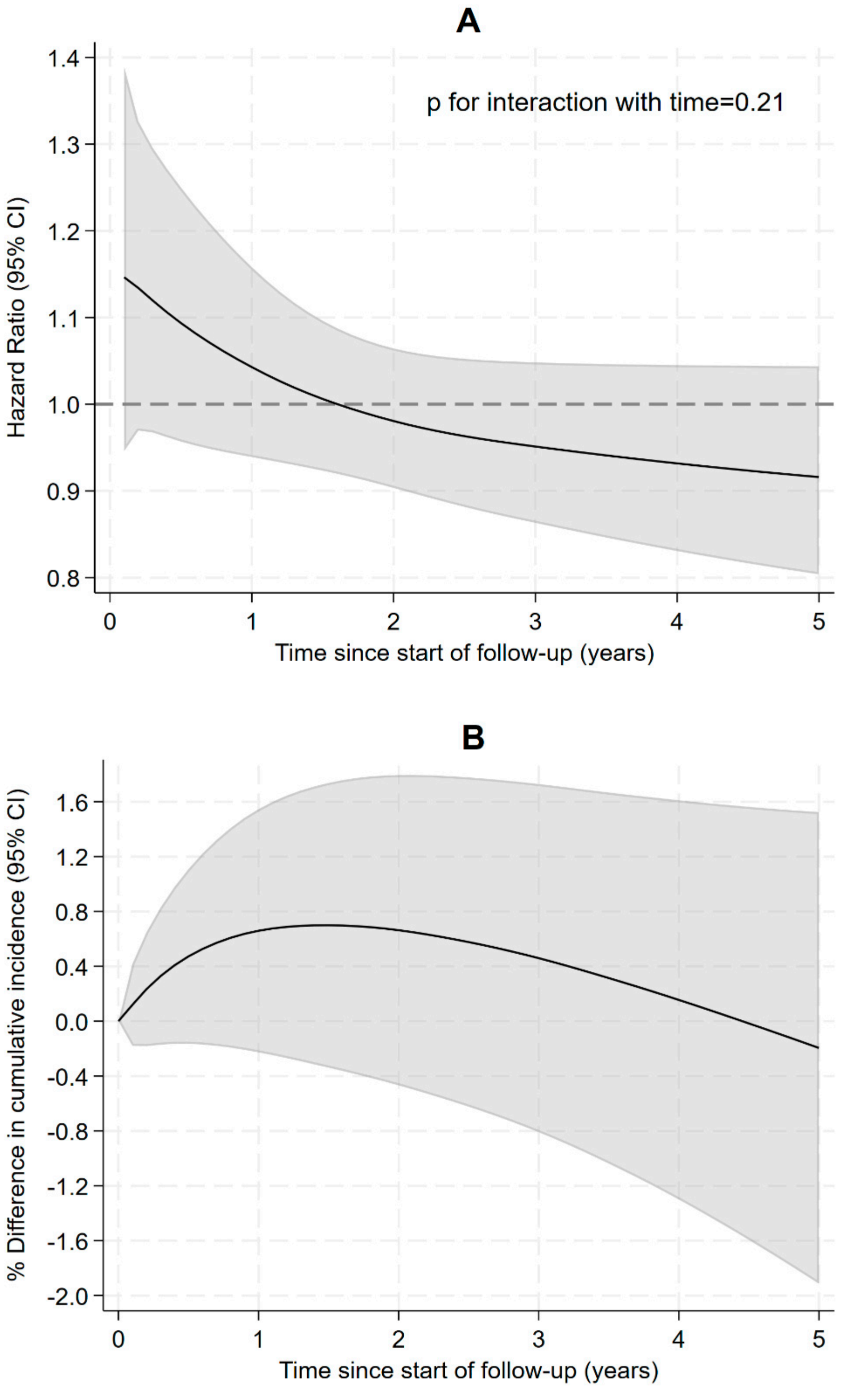

Figure S1. Effect of vitamin D supplementation on incident hypertension.

Panel A shows the time-varying hazard ratio and panel B shows the difference in the cause-specific standardized cumulative incidence.

Estimates (vitamin D versus placebo) are from a flexible parametric survival model that included randomization group, age, sex, state of residence at baseline, and an interaction between randomization group and follow-up time. The interaction between randomization group and follow-up time was assessed using a likelihood ratio test that compared models with and without the interaction term. Cause-specific cumulative incidence probabilities were standardized to the distribution of age, sex, and state of residence at baseline in the entire cohort, and death without prior use of hypertension medication was treated as a competing risk. The difference in cumulative incidence is expressed as a percentage.

Abbreviation: CI, confidence interval

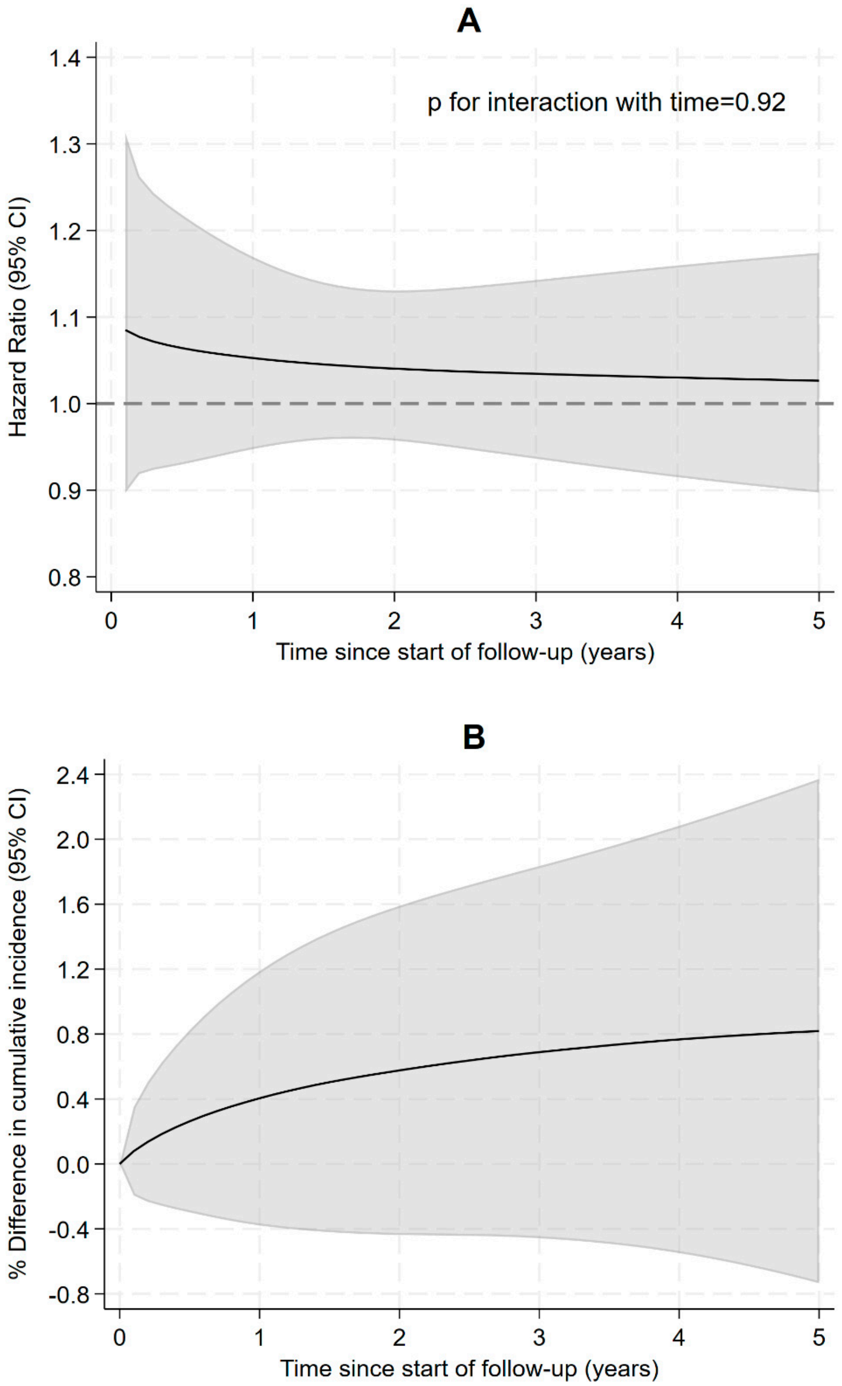

Figure S2. Effect of vitamin D supplementation on incident hypercholesterolemia. Panel A shows the time-varying hazard ratio and panel B shows the difference in the cause-specific standardized cumulative incidence functions.

Estimates (vitamin D versus placebo) are from a flexible parametric survival model that included randomization group, age, sex, state of residence at baseline, and an interaction between randomization group and follow-up time. The interaction between randomization group and follow-up time was assessed using a likelihood ratio test that compared models with and without the interaction term. Cause-specific cumulative incidence probabilities were standardized to the distribution of age, sex, and state of residence at baseline in the entire cohort, and death without prior use of lipid-modifying agents was treated as a competing risk. The difference in cumulative incidence is expressed as a percentage.

Abbreviation: CI, confidence interval

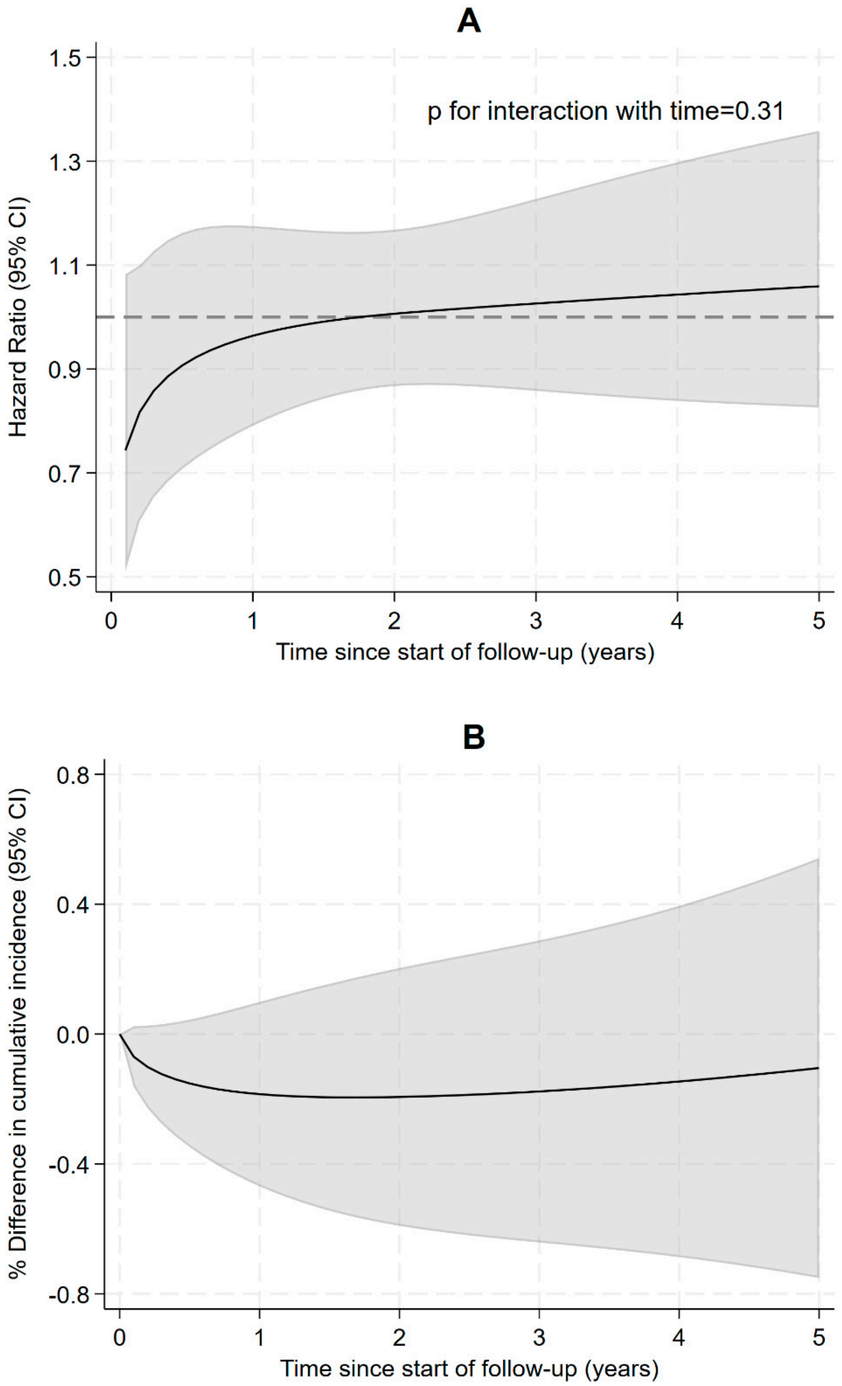

Figure S3. Effect of vitamin D supplementation on incident type 2 diabetes.

Panel A shows the time-varying hazard ratio and panel B shows the difference in the cause-specific standardized cumulative incidence functions

Estimates (vitamin D versus placebo) are from a flexible parametric survival model that included randomization group, age, sex, state of residence at baseline, and an interaction between randomization group and follow-up time. The interaction between randomization group and follow-up time was assessed using a likelihood ratio test that compared models with and without the interaction term. Cause-specific cumulative incidence probabilities were standardized to the distribution of age, sex, and state of residence at baseline in the entire cohort, and death without prior use of diabetes medication was treated as a competing risk. The difference in cumulative incidence is expressed as a percentage.

Abbreviation: CI, confidence interval

	Vitamin D	Placebo	Hazard Ratio (95% CI)	P Value for Interaction
	Incidence Rate per 1000 person-years			
**All participants**	62.4	62.1	1.00 (0.93, 1.08)	
**Age**				**0.32**
< 70 years	52.6	54.4	0.97 (0.87, 1.07)	
≥ 70 years	77.2	73.8	1.04 (0.94, 1.17)	
**Sex**				**0.87**
Men	66.1	66.3	1.00 (0.90, 1.11)	
Women	58.5	57.6	1.01 (0.90, 1.13)	
**Body mass index**				**0.45**
< 25 kg/m²	47.5	49.8	0.95 (0.83, 1.09)	
25 to < 30 kg/m²	67.5	63.4	1.06 (0.94, 1.19)	
≥ 30 kg/m²	80.2	83.3	0.97 (0.84, 1.13)	
**Predicted 25(OH)D concentration**				**0.50**
< 50 nmol/L	65.9	62.8	1.05 (0.90, 1.23)	
≥ 50 nmol/L	61.4	61.8	0.99 (0.91, 1.08)	
**Prevalent hypercholesterolemia**				**0.55**
No	58.3	59.0	0.98 (0.90, 1.07)	
Yes	76.0	72.9	1.03 (0.89, 1.20)	

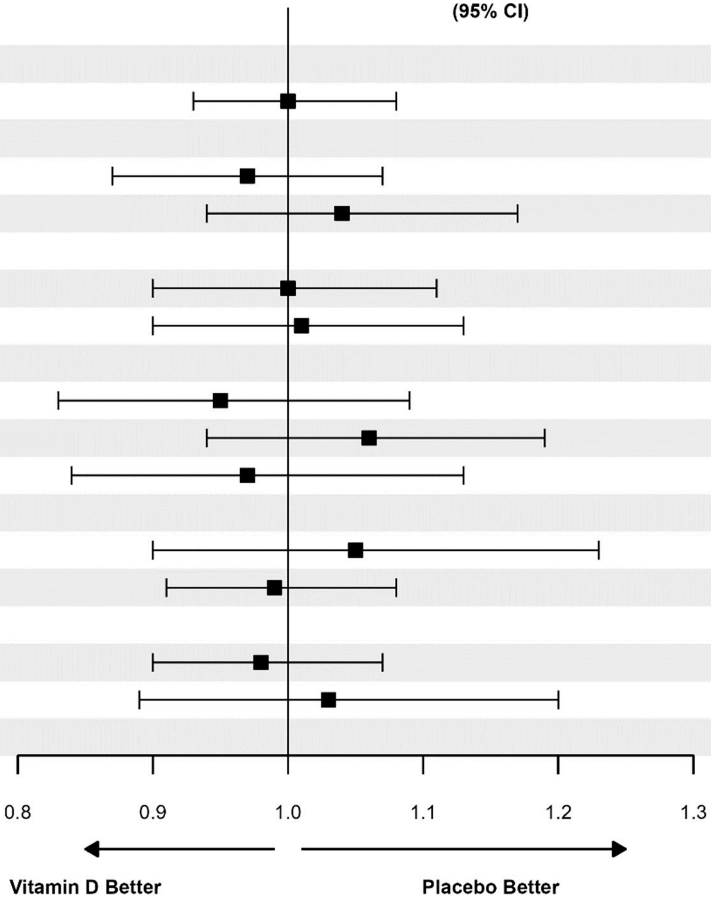

Figure S4. Effect of vitamin D supplementation on incident hypertension overall and within participant subgroups

Hazard ratios estimated using flexible parametric survival models. All models included randomization group, age, sex, and state of residence at baseline. Models producing estimates by levels of age, sex, body mass index, predicted 25(OH)D concentration, and prevalent hypercholesterolemia included the characteristic of interest and an interaction between randomization group and the characteristic of interest. P value for interaction is from a likelihood ratio test comparing models with and without the interaction term. Abbreviation: CI – confidence interval

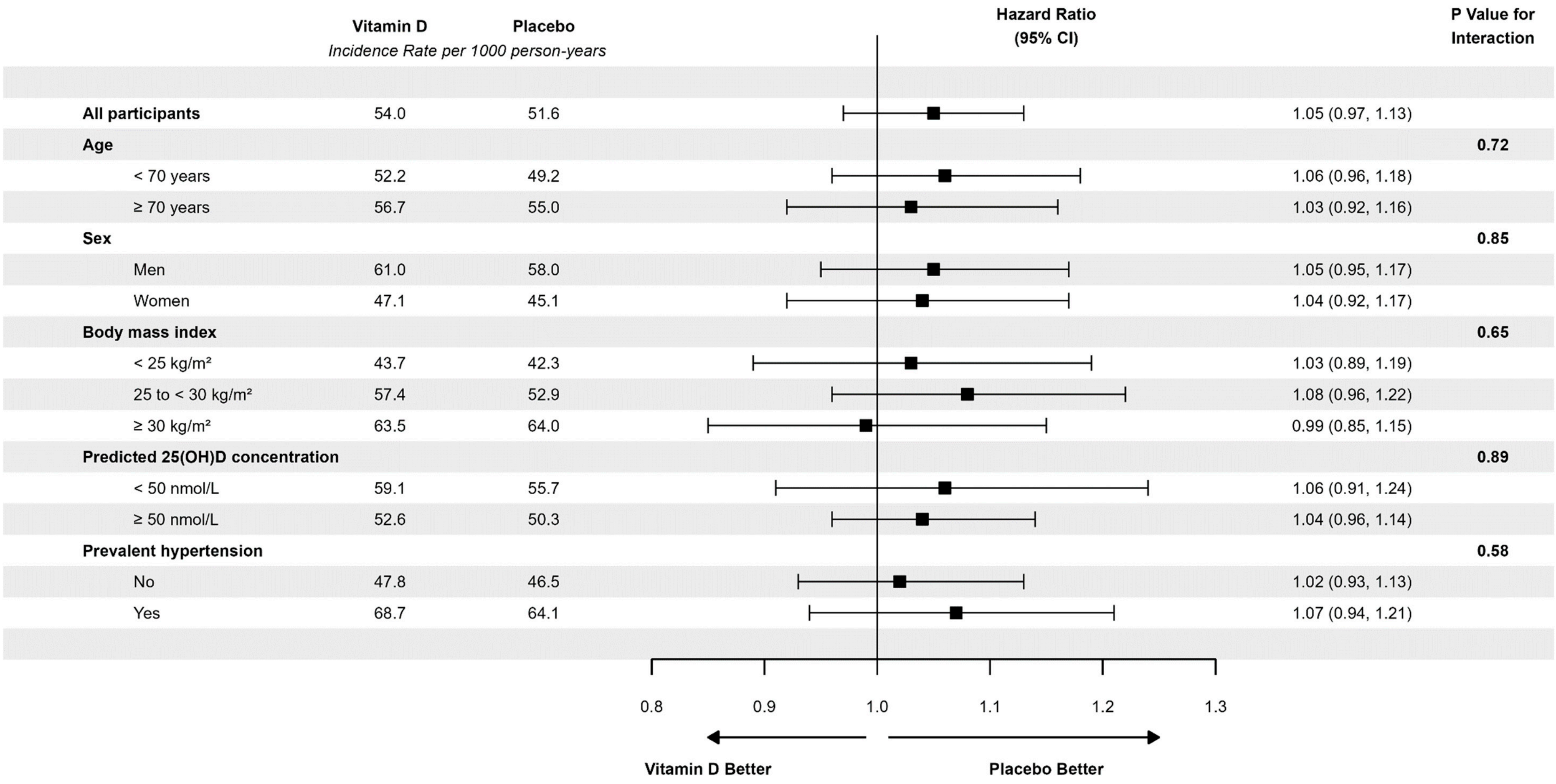

Figure S5. Effect of vitamin D supplementation on incident hypercholesterolemia overall and within participant subgroups

Hazard ratios estimated using flexible parametric survival models. All models included randomization group, age, sex, and state of residence at baseline. Models producing estimates by levels of age, sex, body mass index, predicted 25(OH)D concentration, and prevalent hypertension included the characteristic of interest and an interaction between randomization group and the characteristic of interest. P value for interaction is from a likelihood ratio test comparing models with and without the interaction term. Abbreviation: CI – confidence interval

	Vitamin D	Placebo	Hazard Ratio (95% CI)	P Value for Interaction
	Incidence Rate per 1000 person-years			
**All participants**	9.78	10.0	0.97 (0.84, 1.12)	
**Age**				**0.89**
< 70 years	9.19	9.32	0.98 (0.80, 1.20)	
≥ 70 years	10.4	10.8	0.96 (0.79, 1.17)	
**Sex**				**0.59**
Men	12.0	12.7	0.94 (0.79, 1.12)	
Women	7.28	7.10	1.02 (0.80, 1.30)	
**Body mass index**				**0.43**
< 25 kg/m²	4.20	3.97	1.06 (0.72, 1.56)	
25 to < 30 kg/m²	7.52	8.71	0.86 (0.68, 1.09)	
≥ 30 kg/m²	21.4	20.5	1.04 (0.85, 1.26)	
**Predicted 25(OH)D concentration**				**0.18**
< 50 nmol/L	13.3	11.7	1.13 (0.87, 1.47)	
≥ 50 nmol/L	8.74	9.52	0.92 (0.77, 1.08)	
**Prevalent hypertension**				**0.23**
No	6.83	6.39	1.07 (0.85, 1.33)	
Yes	14.1	15.7	0.90 (0.75, 1.07)	
**Prevalent hypercholesterolemia**				**0.18**
No	7.27	6.83	1.06 (0.87, 1.30)	
Yes	14.6	16.6	0.88 (0.72, 1.06)	

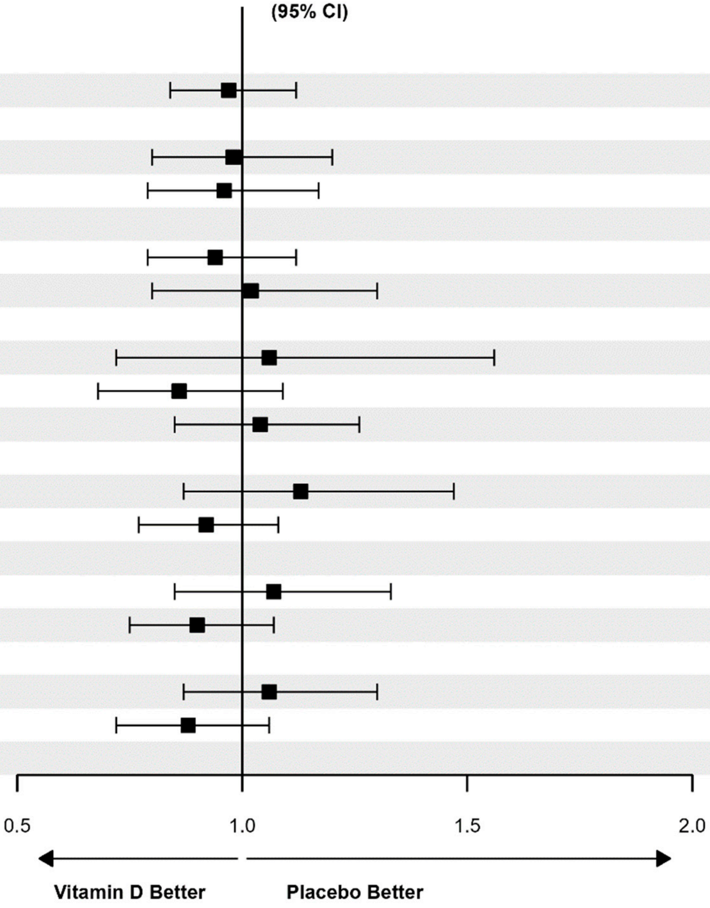

Figure S6. Effect of vitamin D supplementation on incident type 2 diabetes overall and within participant subgroups

Hazard ratios estimated using flexible parametric survival models. All models included randomization group, age, sex, and state of residence at baseline. Models producing estimates by levels of age, sex, body mass index, predicted 25(OH)D concentration, prevalent hypertension and prevalent hypercholesterolemia included the characteristic of interest and an interaction between randomization group and the characteristic of interest. P value for interaction is from a likelihood ratio test comparing models with and without the interaction term. Abbreviation: CI – confidence interval

References

1. WHO Collaborating Centre for Drug Statistics Methodology. ATC/DDD Index 2025. Available online: https://atcddd.fhi.no/atc_ddd_index/ (accessed on 10 March 2023).
